# Simultaneous Measurement of Tricarboxylic Acid Cycle Intermediates in Different Biological Matrices Using Liquid Chromatography–Tandem Mass Spectrometry; Quantitation and Comparison of TCA Cycle Intermediates in Human Serum, Plasma, Kasumi-1 Cell and Murine Liver Tissue

**DOI:** 10.3390/metabo10030103

**Published:** 2020-03-12

**Authors:** Ramji Rathod, Bharat Gajera, Kenneth Nazir, Janne Wallenius, Vidya Velagapudi

**Affiliations:** 1 Metabolomics Unit, Institute for Molecular Medicine Finland (FIMM), HiLIFE, University of Helsinki, Tukholmankatu 8, Biomedicum 2U, 00290 Helsinki, Finland; ramayurnish@gmail.com (R.R.); bngajera@yahoo.co.in (B.G.); Kenneth.nazir@helsinki.fi (K.N.); janne.wallenius@helsinki.fi (J.W.); 2 Fungal Genetics and Biotechnology, Department of Microbiology, University of Helsinki, Biocenter 1, Viikinkaari 9, 00790 Helsinki, Finland

**Keywords:** tricarboxylic acid (TCA) cycle, mass spectrometry, chromatography, metabolites, bioanalytical method validation

## Abstract

The tricarboxylic acid (TCA) cycle is a central part of carbon and energy metabolism, also connecting to glycolysis, amino acid, and lipid metabolism. The quantitation of the TCA cycle intermediate within one method is lucrative due to the interest in central carbon metabolism profiling in cells and tissues. In addition, TCA cycle intermediates in serum have been discovered to correspond as biomarkers to various underlying pathological conditions. In this work, an Liquid Chromatography-Mass Spectrometry/Mass Spectrometry-based quantification method is developed and validated, which takes advantage of fast, specific, sensitive, and cost-efficient precipitation extraction. Chromatographic separation is achieved while using Atlantis dC18 2.1 mm × 100 mm, particle size 3-μm of Waters column with a gradient elution mobile phase while using formic acid in water (0.1% *v*/*v*) and acetonitrile. Linearity was clearly seen over a calibration range of: 6.25 to 6400 ng/mL (r^2^ > 0.980) for malic acid; 11.72 to 12,000 ng/mL (r^2^ > 0.980) for cis-aconitic acid and L-aspartic acid; 29.30 to 30,000 ng/mL (r^2^ > 0.980) for isocitric acid, l-serine, and l-glutamic acid; 122.07 to 125,000 ng/mL (r^2^ > 0.980) for citric acid, glycine, *oxo*-glutaric acid, l-alanine, and l-glutamine; 527.34 to 540,000 ng/mL (r^2^ > 0.980) for l-lactic acid; 976.56 to 1,000,000 ng/mL (r^2^ > 0.980) for d-glucose; 23.44 to 24,000 ng/mL (r^2^ > 0.980) for fumaric acid and succinic acid; and, 244.14 to 250,000 ng/mL (r^2^ > 0.980) for pyruvic acid. Validation was carried out, as per European Medicines Agency (EMA) “guidelines on bioanalytical method validation”, for linearity, precision, accuracy, limit of detection (LOD), limit of quantification (LLOQ), recovery, matrix effect, and stability. The recoveries from serum and tissue were 79–119% and 77–223%, respectively. Using this method, we measured TCA intermediates in serum, plasma (NIST 1950 SRM), and in mouse liver samples. The concentration found in NIST SRM 1950 (*n* = 6) of glycine (246.4 µmol/L), l-alanine (302.4 µmol/L), and serine (92.9 µmol/L).

## 1. Introduction

The tricarboxylic acid (TCA) cycle or Krebs’ cycle consists of a set of responses active under aerobic conditions aiming to produce energy in the form of Adenosine triphosphate (ATP). In adding, the TCA cycle delivers precursors to biosynthesis of certain amino acids and Reduced Nicotinamide Adenine Dinucleotide (NADH) [1]. Being part of central carbon metabolism the interest in quantifying TCA cycle metabolites (Supplementary Figure S1) is apparent, e.g., metabolic dependencies and development of cancer cells or tumor tissues [2,3]. In recent years, the alterations in TCA cycle compounds that are present in serum have also been investigated to correspond to various underlying pathological or physiological conditions. The TCA cycle metabolites have been proposed as biomarkers in serum for e.g., cardiovascular risks [4], leukemia [5], nasopharyngeal carcinoma [6], prostate cancer [7], diabetic nephropathy [8], and nutritional state [9].

To date, multiple mass spectrometry-based approaches are able to quantitate TCA-cycle related compounds utilizing various chromatographic techniques, such as gas chromatography using acylation or silylation of analytes, capillary electrophoresis, and liquid chromatographic (LC) approaches that are based on ion exchange/exclusion, ion pairing, hydrophilic interaction, or reverse phase. Tan et al. previously covered the variety of literature examples, with their pros and cons [10]. Many of the TCA cycle molecules are short chain carboxylic acids, the quantitation of which is generally challenged by bad retaining in reverse phase chromatographic methods, less sensitivity because of ionization efficiency, and more suppression of ion using electrospray ionization (ESI) [11]. While considering workflow fluency for high-throughput analysis and maintaining high precision, various challenges are present in different approaches, and analysis from blood fluid introduces additional complexity in the form of matrix effect and ion suppression [12]. Commonly, high variation between replicates can be observed if no proper internal standard is used, especially inter-samples [13,14].

The traditional approaches to serum pretreatment for Liquid Chromatography-Mass Spectrometry (LC-MS) analysis comprise phospholipid removal plates, protein precipitation, and liquid-liquid extraction (LLE) [15]. The presence of endogenous phospholipids in the serum matrix can cause a matrix effect and ion suppression in the LC-MS methods [16,17]. A readymade sample filter cartridge provides a cost-efficient approach to remove the phospholipids and precipitate proteins for compound analysis.

This work presents the development and validation for a method to quantify the TCA cycle related intermediate compounds with targeted LC-MS/MS in human serum, plasma, kasumi-1 cell, and murine liver tissue. The method combines protein precipitation with the use of a specific corresponding ^13^C isotope internal standard for each analyte, providing a cost efficient method and fast pretreatment with the potential to provide high-throughput analysis with 96-well plates. The pretreatment was matched with the readily compatible fast reverse-phase based chromatographic method. Additional preparation steps, e.g., the evaporation of extraction solvent and reconstitution to eluent, were bypassed, therefore reducing the organic phase by dilution with the aqueous mobile phase in order to enhance chromatography [18].

## 2. Results and Discussion

### 2.1. Method Development

The (Ultra Performance Liquid Chromatography Mass Spectrometry/Mass Spectrometry (UPLC MS/MS) method was developed with the use of positive and negative ESI ionization mode. Mass range of Xevo-TQS triple quadrupole mass spectrometer is 2–2048 *m*/*z*. This instrument was calibrated with solution having masses from 62.9636 to 1764.592, but there is no calibration reference points near low mass (2 *m*/*z*) or near high (2048 *m*/*z*). This instrument is not high resolution MS and you cannot really see any difference and the mass accuracy will be same. Calibration only done in positive mode and negative mode will use positive mode calibration and mass accuracy. The product ion selection producing the highest response further makes the selectivity and sensitivity of the method better. There difference between Glutamine (147.1 *m*/*z*) and Glutamic acid (148.1 *m*/*z*) is of 1 *m*/*z*. Glutamine has five carbon atoms, which means that 5% of the carbons are C13 and 95% are C12. The 5% will be the amount of interference have on the glutamic acid channel, as it is not chromatographically separate from them and the same will uniformly apply to all sample and standards, so overall there were no more significant impacts on outcome. However, in pyruvic acid and lactic acid there is difference of 2 *m*/*z*, so there is considerably less chance of interference because difference of 2 *m*/*z* will be separate even from isotopic mass range. Various stationary-phases, like Discovery HS FS-3, 3um, 2.1 × 150 mm (no proper separation between Citric acid and Iso citric acid and peak shapes at lower levels of Glutamic acid, pyruvic acid, Lactic acid, Fumaric acid was poor due to less response and tailing), ACQUITY UPLC BEH Amide, 2.1 mm × 100 mm, 1.7 µm (poor peak shape for few analytes was observed with tailing) and mobile-phase compositions (ranging from 10 mM ammonium formate (10 mM ammonium acetate) pH 4: 0.1% Formic acid in Acetonitrile and methanol, 0.1% formic acid in water: Acetonitrile were tested, and, at last, the most optimal separation, peak shape, and response were obtained in an Atlantis dC18 2.1 × 100 mm, 3-μ column with 0.1% formic acid in water (*v*/*v*), and an acetonitrile as mobile phase in the gradient elution mode. The stability of standard solutions stored at −20 °C was evaluated over a one-month period. Acetonitrile in 1% formic acid was used as extraction solution, which was prone to protein precipitation in plasma and compatible with Waters Ostro 96 well plate 25 mg (Part No. 186005518). This plate serves as two sample extraction techniques i.e., Protein precipitation and solid phase extraction and the solvent recommended for this plate is 1% Formic acid in Acetonitrile, which is why extraction solvent is taken as 1% Formic acid in Acetonitrile. However, the final reconstitution solution was selected as 0.1% formic acid in water because extraction solvent i.e., 1% Formic acid in Acetonitrile as reconstitution solution is prone to broad peak shape of few metabolites. In tissue, second extraction solvent (acetonitrile:water, 90:10, 1% formic acid) was additionally used, because, by increasing water portion of the extraction solvent, it could extract higher metabolites. Accordingly, this step is just an additional step in tissue to obtain more metabolite extract from complex tissue sample. Cleaner extracts, good recovery, and low matrix effect for the biological matrix was observed with the above plate and acetonitrile in 1% formic acid as extraction solvent. An isotope-labeled internal standard was used to accurate determine any possible loss of metabolites throughout sample preparation. When using the extraction method that is described above, the matrix effect was found less often and no affect was found on the results due to the structural match of the internal standard for almost all metabolites.

### 2.2. Method Validation

#### 2.2.1. Selectivity and Specificity

**Figure 1:**
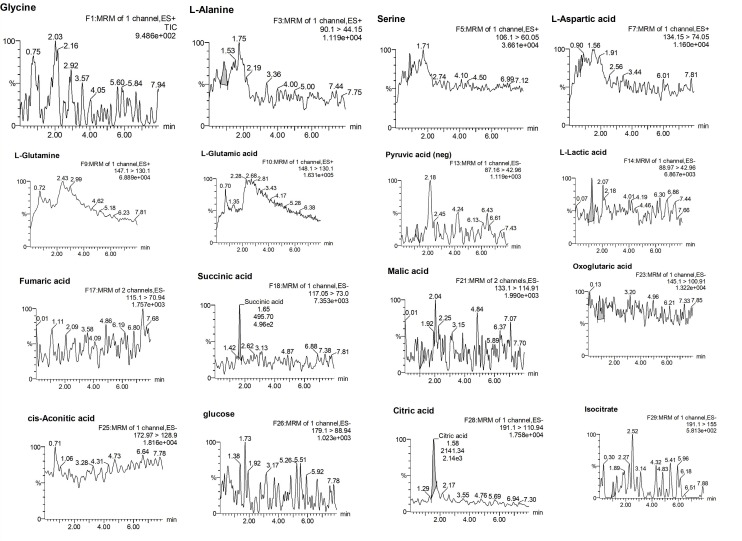
Blank chromatogram of tricarboxylic acid (TCA) cycle intermediate.

**Figure 2:**
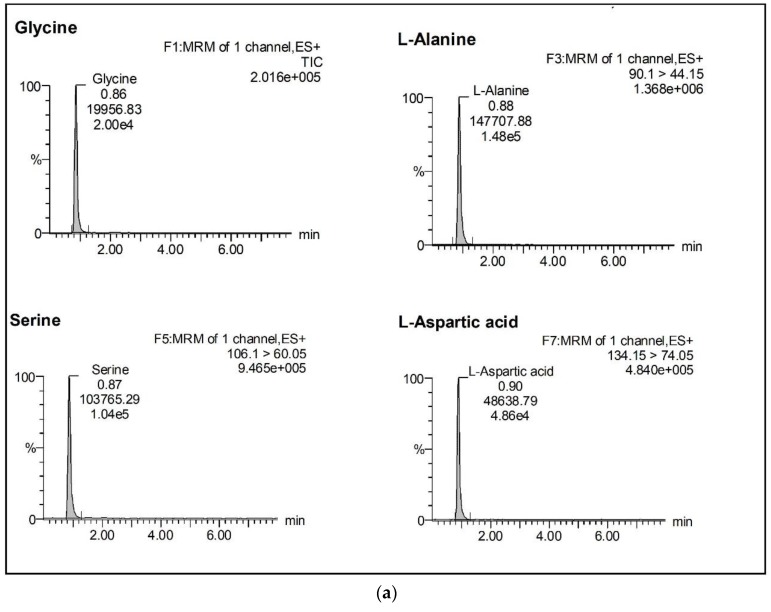

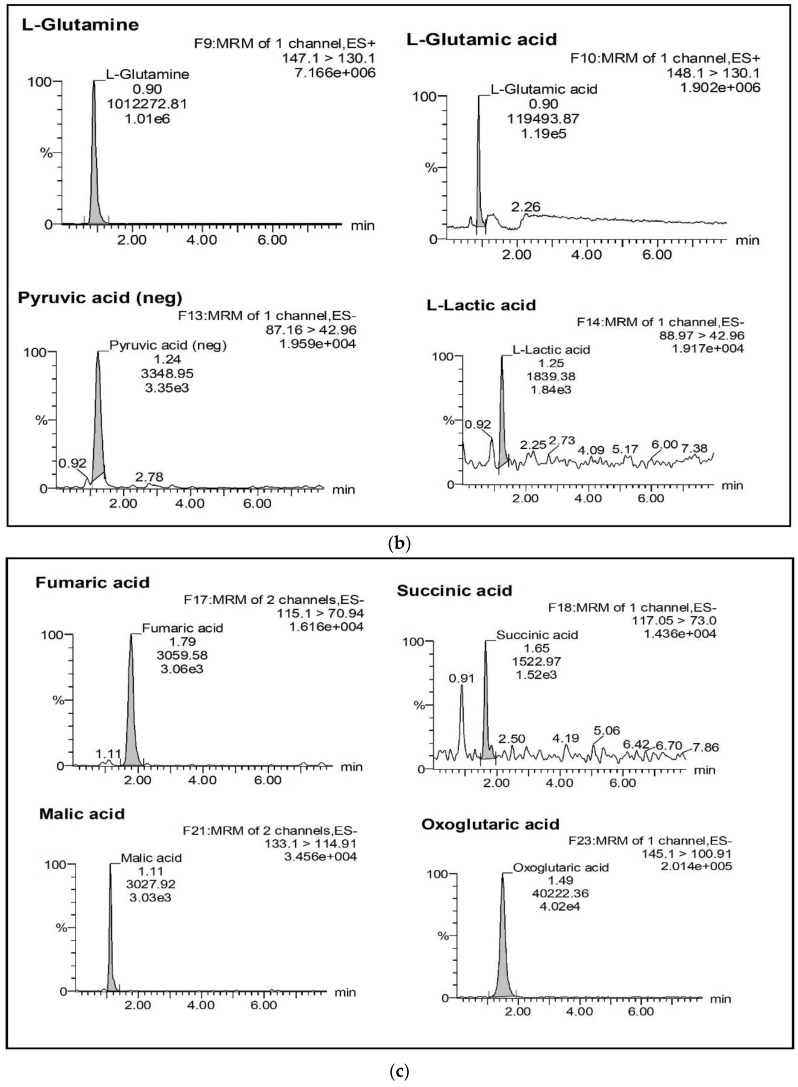

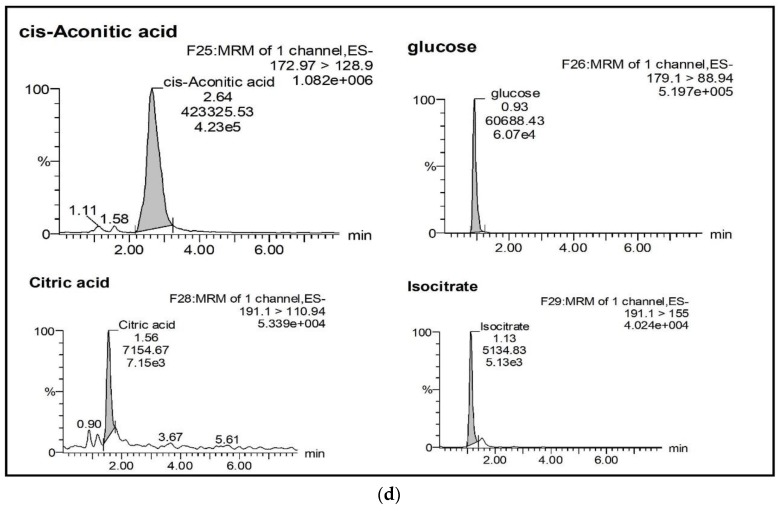
(**a**) Chromatogram of TCA cycle intermediate; (**b**) Chromatogram of TCA cycle intermediate; (**c**) Chromatogram of TCA cycle intermediate; and, (**d**) Chromatogram of TCA cycle intermediate.

There were no significant interference peaks from the matrix components in their respective retention-time windows, which indicated the acceptability of selectivity and specificity of the TCA cycle intermediates in our method. Each tissue, cell, and serum-sample was injected by repeating five times and every peak was confirmed to have only eluted from the target analyte, which indicated that they are specific to their corresponding MRM transitions. Figure 1 and Figure 2 provide the chromatograms of the entire 16 TCA cycle intermediates of Blank and QC sample (refer to Supplementary Figure S2).

#### 2.2.2. Auto-Sampler Carryover

In general, for the majority of the TCA cycle intermediates, there was no significant carry over observed in the blank sample following the high concentration standard (Level 11), which is no greater than 20% of the lowest standard concentration (Level 1) and 5% for the internal standard. Serine, lactic acid, and citric acid have carryover of 18%, but it is within the limit. The carryover of succinic acid was more than 20%. No carryover was observed in the internal standards. Therefore, we can conclude that the syringe, needle, column, and seal washes were adequate to avoid any sample carryover.

#### 2.2.3. Linearity, Accuracy, and Precision

Each calibration curve was statistically evaluated with respect to slope, intercept, and constructed while using appropriate and best-fit regression models and 1/x^2^ weighing factors. The observed coefficient of determination (r^2^) value for individually TCA cycle intermediate was higher than 0.99. The accuracy and precision (within and between (two different days) run) evaluated by measuring the difference in back-calculated concentration and nominal concentration of calibration curve standards and quality control samples (*n* = 6 replicates) at higher, middle, lower, and lower limit of quantification. The percent mean accuracy and coefficient of variance in all three batches were found within 20% for the major TCA cycle intermediates, except for malic acid (22%) and glutamic acid (26%) (Table 1 and Table 2).

#### 2.2.4. Recovery and Matrix Effect

For TCA cycle intermediates, the recoveries are between 73 to 137% in serum, 50 to 172% in tissue (liver) (except 250% in cis aconitic acid), and 94 to 120% in cell, with worthy repeatability at all three (low, medium, and high) concentrations levels. The results of recovery obtained at each QC levels in serum, cell, and tissue are given in supplementary below for further reference. However, the Coefficient of Variance of recoveries at these three concentration levels was within 17% in serum, cell, and tissue (murine liver), except for pyruvic acid, succinic acid, and cis aconitic acid with CV 22%, and oxo-glutaric acid and aspartic acid with CV 50% in tissue (murine liver) (Table 2). The result data are available in Supplementary Table S1.

The matrix effects were within −2 to 2 (less than 1 indicates ion suppression and more than 1 indicates ion enhancement) for most TCA Cycle intermediates in serum, cell, and tissues at the HQC level. At MQC level values that were within the range of −2 to 2 for cell, serum, and some metabolites in tissue, except within 3 (serine, lactic acid), within 4 (cis-aconitic acid, isocitric acid). At LQC level values within the range of −2 to 2 for cell, some metabolites in serum and some metabolites in tissue, except within the range of 5 in serum (glycine, Alanine, l-aspartic acid, l-Serine, l-Glutamine, l-Glutamic acid, l-Lactic acid, and Malic acid) and 5 to 10 in tissue (l-Serine, l-Glutamine, l-Glutamic acid, l-Lactic acid, Fumaric acid, Succinic acid, Malic acid, and Citric acid). To reduce the matrix effect, used individual isotope-labeled internal standards for respective each individual TCA cycle intermediate analyte. In the present work, 14 isotopic labeled internal standards were used for 16 TCA cycle intermediates. The result data are available in Supplementary Table S1.

#### 2.2.5. Stability

The stock solutions and working solution of TCA cycle intermediates were freshly prepared during individual LC-MS/MS runs. Though, the stability was assessed at the HQC and LQC level by storing the TCA cycle intermediates at −20 °C for seven days and subsequently carrying out the analysis. During the storage, no loss of analyte was observed, because the degradation was within 15% in stock and working solution. This is in harmony with work that was done by Keevil and colleagues, reporting that citric acid was stable at −20 °C for 30 days [3]. The autosampler stability was assessed at the HQC and LQC level by storing the extracted sample at 4 °C for 48 h. During the storage at 4 °C for 48 h, no loss of analyte was observed, because the degradation was within 15% in extracted sample. The CV (%) value was <10% for analyte in this study (Table 3). Cell and tissue are available in a wide range of species, so it is difficult to identify the ideal species for long term in matrix and freeze thaw stability experiment. That’s why not presented here.

#### 2.2.6. Limit of Detection and Limit of Quantification

We calculated the Limit of detection values of the TCA Cycle Intermediates by means of a signal-to-noise ratio of 3 (Table 1). The lowest calibration standard (Level 1) with accepted accuracy and precision was considered as LOQ (Table 1).

### 2.3. Biological Sample Analysis

**Figure 3:**
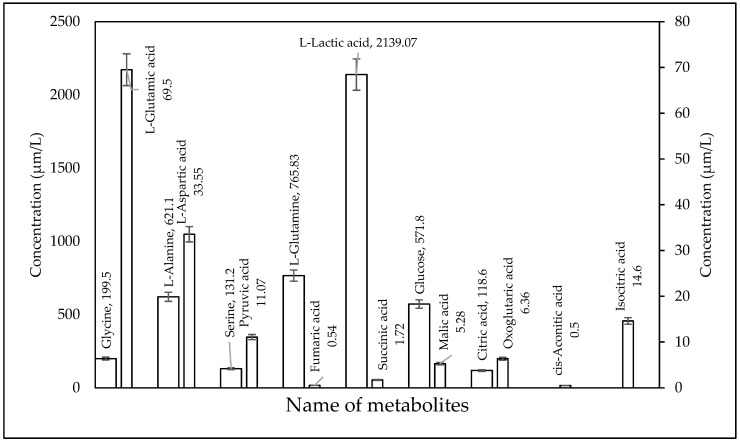
Measure of TCA cycle intermediates concentration in six Quality Control human serum.

**Figure 4:**
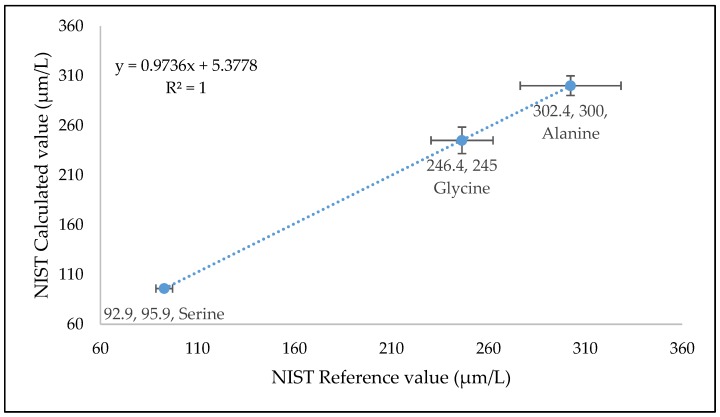
Measure of TCA cycle intermediates (Glycine, Alanine, and Serine) concentration in National Institute of Standards Technology, Standard Reference Material, Plasma 1950. The error bar is in standard deviation. Calculated value is the concentration obtained by injecting six replicate NIST SRM 1950 samples by this method and the reference value is the value given in the certificate of NIST SRM 1950.

**Figure 5:**
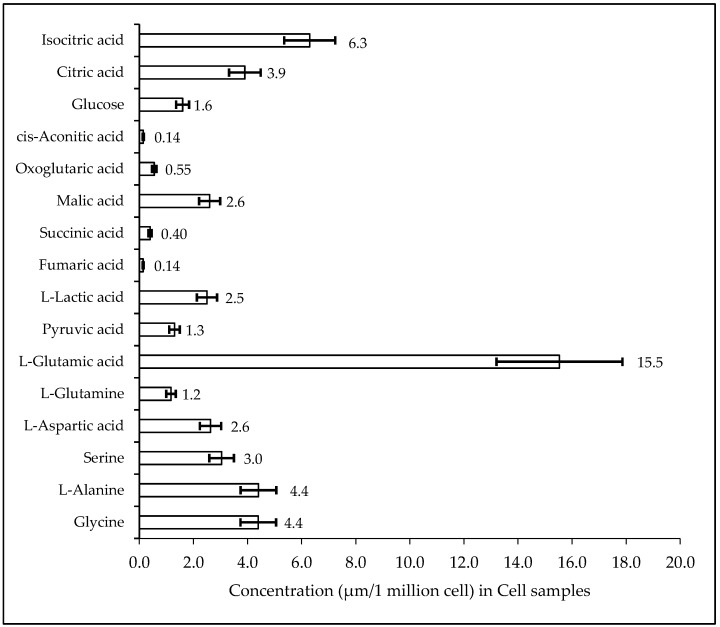
Measure of TCA cycle intermediate concentration in Kasumi-1 Cell line.

**Figure 6:**
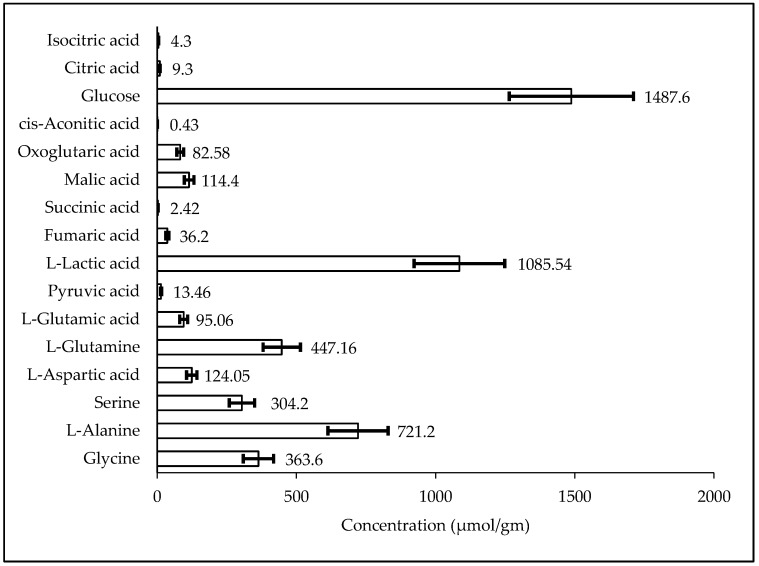
Result of TCA cycle intermediate in murine liver tissue.

The validated method was used for quantification of TCA Intermediate analytes in human serum QCs (*n* = 6), plasma NIST SRM, kasumi-1 cell, and murine liver tissue. Figure 3, Figure 4, Figure 5, and Figure 6 shows the quantified analyte amounts. Lactic acid and glucose micro molar concentrations were approximately 10–100 times higher in scale compared to the other analytes in serum, plasma, and murine liver tissue. The concentration found in NIST SRM 1950 (*n* = 6) of glycine (246.4 µmol/L, Std Dev: 13.29), l-alanine (302.4 µmol/L, Std Dev: 9.88), and serine (92.9 µmol/L, Std Dev: 2.25) are within the limit of reference values, i.e., 245 ± 16 µmol/L, Std Dev: 16 for glycine, 300 ± 26 µmol/L, Std Dev: 26 for l-alanine, and 95.9 ± 4.3 µmol/L, Std Dev: 4.3 for serine.

The average concentrations that were obtained from serum samples for citric acid (118.6 ± 1.1 µmol/L) were comparable to previous reports using a quantitative GC–MS assay with the derivatization of citric acid from normal human sera (102.28 ± 37.5 µmol/L) [19]. Previous studies confirmed the µmol/L concentration in the serum of 30 healthy individuals for L-alanine (588 ± 216.6 µmol/L), l-serine (105.4 ± 21.2 µmol/L), and glycine (93.28 ± 19.86 µmol/L) [20] and the comparative concentration from serum was 621.1 ± 158.1, 132.2 ± 15.1, and 199.5 ± 5 µmol/L, respectively.

The kasumi-1 Cell line samples were analyzed and the concentration in one-million cells of metabolites are as glycine (4.4 µmol), alanine (4.4 µmol), serine (3.0 µmol), aspartic acid (2.6 µmol), glutamine (1.2 µmol), glutamic acid (15.5 µmol), pyruvic acid (1.3 µmol), lactic acid (2.5 µmol), fumaric acid (0.14 µmol), succinic acid (0.40 µmol), malic acid (2.6 µmol), oxoglutaric acid (0.55 µmol), cis-aconitic acid (0.14 µmol), glucose (1.6 µmol), citric acid (3.9 µmol), and isocitric acid (6.3 µmol).

## 3. Materials and Methods

### 3.1. Chemicals, Reagents and Samples

Tricarboxylic acid standards [*cis*-aconitic acid, citric acid (≥99%), fumaric acid (≥99%), isocitric acid (≥93%), malic acid (≥99%), pyruvic acid, oxo-glutamic acid (≥99%), glycine, l-alanine, l-serine, l-aspartic acid, l-glutamine, l-glutamic acid, l-lactic acid, succinic acid (≥99%) and d-glucose] were purchased from Sigma^®^ (M/s Sigma-Aldrich Company Ltd., Dorset, UK). L-malic acid (^13^C_4_, 99%), citric acid (^13^C_6_), l-aspartic acid (U-^13^C_4_), l-serine (^13^C_3_), l-glutamic acid (^13^C_5_), glycine (1,2-^13^C_2_), alpha-ketoglutaric acid (^13^C_5_), l-alanine (^13^C_3_), l-glutamine (^13^C_5_), sodium l-lactate (^13^C_3_), d-glucose (U-^13^C_6_), fumaric acid (^13^C_4_), succinic acid (^13^C_4_), and sodium pyruvate (^13^C_3_) was purchased from M/s Cambridge Isotope Laboratories, Inc. (M/s CK Isotopes Ltd., Leicestershire, UK). LC–MS grade acetonitrile, formic acid, and methanol were purchased from Sigma (M/s Sigma-Aldrich Company Ltd., Dorset, UK). Deionized water (18 MΩ·cm at 25 °C) that was used for solution preparation was made while using a Milli-Q water purification system (Bamstead EASYpure RoDi ultrapure water purification system, M/s Thermo scientific, Waltham, OH, USA). Precellys 2mL homogenizing tubes with 2.8 mm ceramic beads and reinforced tube were purchase from M/s VWR International (Helsinki, Finland). The whole blood from which serum was prepared during method optimization and validation was obtained from the M/s Finnish Red Cross blood service (Helsinki, Finland). Dr. Emmy Verschuren’s laboratory kindly donated the murine liver tissue (FIMM, HiLife, Biomedicum 2, University of Helsinki, Finland). Our research collaborators Dr. Mahesh Tambe donated the Kasumi-1 Cell line samples (FIMM, HiLife, Biomedicum 2, University of Helsinki, Finland). NIST standard reference material (SRM) 1950 plasma was purchased from M/s Sigma-Aldrich (Gillingham, UK). All of the biological samples were stored at −80 °C. The details on materials are available in Supplementary Table S1.

### 3.2. Solution Preparation

The stock solutions for individual metabolite were prepared at a concentration of 10 mg/mL (except d-Glucose 100 mg/mL). An intermediate solution covering all metabolites with a different concentration for individually was prepared in 0.1% formic acid in water. The calibration curve solutions with 11 Levels were prepared by serial dilution from the intermediate solution in 0.1% formic acid in water to yield a concentration range of: 6.25 to 6400 ng/mL for malic acid; 11.72 to 12,000 ng/mL for cis-aconitic acid and L-aspartic acid; 29.30 to 30,000 ng/mL for isocitric acid, l-Serine, and l-Glutamic acid; 122.07 to 125,000 ng/mL for citric acid, oxoglutaric acid, glycine, l-alanine, and l-glutamine; 527.34 to 540000 ng/mL for L-lactic acid; 976.56 to 1,000,000 ng/mL for d-Glucose; 23.44 to 24,000 ng/mL for fumaric acid and succinic acid; and, 244.14 to 250,000 ng/mL for pyruvic acid. The QC samples were prepared while using separate stock solutions at high (75% of the upper calibration curve range), medium (nearby to level 6, around 30–50% of the calibration curve range), low (within three times the LLOQ), and lower limit of quantification.

An internal standard dilution that was prepared from 14 different stocks. (l-malic acid (^13^C_4_), citric acid (^13^C_6_), l-aspartic acid (U-^13^C_4_), l-serine (^13^C_3_), l-glutamic acid (^13^C_5_), glycine (1,2-^13^C_2_), alpha-ketoglutaric acid (^13^C_5_), l-alanine (^13^C_3_), l-glutamine (^13^C_5_), sodium l-lactate (^13^C_3_), d-glucose (U-^13^C_6_), fumaric acid (^13^C_4_), succinic acid (^13^C_4_), and sodium pyruvate (^13^C_3_). All of the standard and internal solutions were protected from light and stored at −20 °C. During method development and validation, these solutions are stable and used. The extraction solvent used was 1% formic acid in acetonitrile and the second extraction solvent was 1% formic acid in acetonitrile:water (90:10), which was used for tissue metabolite extraction.

### 3.3. Sample Extraction

#### 3.3.1. Extraction Protocol for Murine Liver Tissue Samples

Approximately 20 mg of frozen tissue was collected in homogenization tubes (Bertin Technologies, Montigny-le-Bretonneux, France), with 2.8 mm ceramic (zirconium oxide) beads and 2 mL standard tube volume. Subsequently, the volume of 20 μL of an internal standard dilution solution was added to the sample and they were equilibrated on ice for 2 min. Subsequently, 300 μL of an 1% formic acid in acetonitrile was added and vortexed for 20 s. The tissue samples were homogenized while using a tissue homogenizer (Bertin Technologies, France) over three cycles of 30 s at 5500 rpm each with a 10-s pause interval between cycles. After homogenization, the samples were centrifuged at 14,000 rpm for 15 min. at 4 °C. Supernatant was loaded into a Waters Ostro 96 well plate 25mg (Part No. 186005518). The same homogenized steps were repeated with a second extraction solvent (acetonitrile:water, 90:10, 1% formic acid). Collected supernatants were passed through a Waters Ostro 96 well plate 25mg (Part No. 186005518) using a vacuum, subsequently 110 μL of filtrate was mixed with 400 μL 0.1% formic acid in water. A volume of 5 μL of was then injected into the LC-MS/MS system.

#### 3.3.2. Extraction Protocol for Serum and Plasma

Approximately 100 μL of serum or plasma loaded into Waters Ostro 96 well plate 25 mg (Part No. 186005518). A volume of 10 μL of ISTD dilution mix was added into the same plate, followed by the addition of 300 μL of 1% formic acid in acetonitrile. The plate was filtered while using a vacuum. Subsequently, 110 μL of filtrate was mixed with 400 μL 0.1% formic acid in water. A volume of 5 μL was injected into the LC-MS/MS system.

#### 3.3.3. Extraction Protocol for Cell

Approximately, in one-million cells, a volume of 90 µL 0.1% Formic acid in water was added and vortexed to mix, and 10 μL of ISTD dilution mix was added. Subsequently, 300 µL of 1% formic acid in acetonitrile was added and vortex to mix. Three cycles of extraction were carried out by vortexing for two minutes and then sonicating for 1 min. (settings: sweep mode, frequency 37, power 60, no heating). After this, the samples were centrifuged at 14,000 rpm for 15 min at 4 °C. Supernatant were loaded into Waters Ostro 96 well plate 25 mg (Part No. 186005518) and pass through it using vacuum. Subsequently, 110 μL of filtrate was mixed with 400 μL 0.1% formic acid in water. A volume of 5 μL was injected into the LC-MS/MS system.

### 3.4. Instrument and Analytical Conditions

Waters Acquity UPLC system coupled to a Xevo-TQS triple quadrupole mass spectrometer equipped with an electrospray ionization probe (M/s Waters Corporation, Milford, MA, USA). An Atlantis dC18 (2.0 × 100 mm, 3-μm particles) reversed-phase analytical column from Waters (M/s Milford, MA, USA) was used as a chromatographic separation column. Gradient elution was carried out with a flow rate of 0.600 mL/min. while using 0.1% formic acid in water as mobile phase A and acetonitrile as mobile phase B. The gradient elution was initiated from 100% to 90% of mobile phase A in 8 min., then 90% to 100% followed from 8 to 8.1 min. and the same is maintained for 11 min. The column oven and auto-sampler temperatures were set to 40 ± 3 °C and 5 ± 3 °C, respectively. Positive and negative ion polarity modes were both used for electrospray ionization. Table 4 provides cone voltage, collision energy, and the MRM transition. The source-dependent parameters were constantly maintained throughout analysis, including capillary voltage (3.5 kV), desolvation temperature (500 °C), desolvation gas flow (600 L/h), cone gas flow (150 L/h), and collision gas flow (0.15 mL/min.). Nitrogen (purity > 99.9%) and argon (purity 99.998%) were used as the desolvation and collision gases, respectively. The MRM transition mode was used for the quantification of metabolites with an individual span time of 0.1 s being taken in their individual MRM transition. The dwell time was automatically calculated based on the region of the retention time window, the number of MRM transition, and the number of data points required to form a peak. Mass Lynx 4.1 software used for data acquisition and integrations. The peak area ratio (area of metabolite/ area of IS) that was used for quantification of the metabolites and weighting factor of 1/x^2^ was used in the fitting of linear least squares calibration curves. 

### 3.5. Validation

Validation of the analytical method was performed to verify the reliability of the developed method for the analysis of a large number of samples. Method validation was carried out according to the European Medicines Agency (EMA) “guidelines on bioanalytical method validation” (EMEA/CHMP/EWP/192217/2009 Rev. 1) [21]. Linearity, precision, accuracy, recovery, matrix effect, stability, limit of detection (LOD), and limit of quantification (LOQ) were evaluated for the developed method. Quality control (QC) samples at high (75% of the upper calibration curve range), middle (around 30–50% of the calibration curve range), low (within three times the LLOQ), and lower limit of quantification concentration levels were prepared by spiking the standard solution in the respective homogenized biological matrices (during recovery, matrix effect QC samples at high, middle, and low levels were spiked in pooled of each matrix type i.e., serum, cell, and tissue) to complete all method validation experiments. However, these matrices have natural endogenous TCA cycle intermediates, thus the concentration of the non-spiked samples were subtracted from the concentration of the spiked samples. Complete validation was carried out for the murine liver tissue and serum. A calibration curve in aqueous diluent was used for method validation. A system suitability experiment was carried out by injecting six consecutive injections of middle quality control concentration at the start of method validation and on every day to check the instrument performance and response reproducibility in terms of the peak area for each metabolite. The coefficient of variation (CV %) for the system suitability was within the range of 15%.

#### 3.5.1. Selectivity and Specificity

The selectivity and specificity for individual metabolite was evaluated by serum-spiked samples (*n* = 6) with a known amount of standard. The selectivity and specificity evaluated by chromatographic interferences from other endogenous compounds of the biological matrix present at the retention time of the target analyte.

#### 3.5.2. Auto-Sampler Carryover

The auto-sampler carryover perform by injecting the highest standard (Level 11) of the TCA cycle intermediates in the calibration curve followed by blank injections and the lowest standard (Level 1) and 5% for the internal standard, as per the European Medical Agency guidelines for bioanalysis.

#### 3.5.3. Linearity, Accuracy and Precision

To assess the linearity, the response of the instrument and concentration of TCA cycle intermediates should be known and evaluated over a specified concentration range. The calibration standards were prepared in the aqueous solution, because the same calibration standards were used for different matrices, i.e., serum, plasma, cell, and tissue. The established range of concentration for all of the analytes of interest allowed for adequate measurement of their normal concentrations present in the validated matrices based on the values that are given in the Human Metabolome database (www.hmdb.ca). Eleven calibration concentration levels were used, in addition to the blank sample and a zero sample (processed with internal standard). The precision and accuracies were assessed with three injection batches on separate days, including six replicates of QC samples at high, medium, and low concentrations, along with calibration curve standards. The calibration curve was plotted by using the peak area response ratios (standard/labeled standard) versus the concentrations of the individual TCA cycle intermediates. Each calibration curve was statistically evaluated and constructed while using appropriate regression models, weighing factors, and transformations. For accuracy and precision, the mean accuracy (%) and coefficient of variance were determined, respectively. 

#### 3.5.4. Recovery and Matrix Effect

The recovery efficiencies for each analyzed TCA cycle intermediate were determined by comparing the responses that were obtained from the spiked QC matrix (serum, cell, and tissue) at high, medium, and low concentrations that were prepared by spiking with a standard QC at high, medium, and low concentrations before and after extraction. The matrix effect (percentage of ion suppression or enhancement of the MS signal) was determined by comparing the response that was obtained from spiked QC matrix (serum, cell, tissue) at high, medium, and low concentrations that were prepared by spiking with a standard QC at high and low concentrations against spike in aqua. Since there were endogenous TCA cycle intermediates, we subtracted the endogenous concentrations from the samples that were spiked. This experiment was performed using six replicates. Recovery and Matrix factor at each QC level in different Matrix was evaluated by the following formula:

(1) $$\mathrm{Recovery}=\frac{\mathrm{Peak}\;\mathrm{area}\;\mathrm{in}\;\mathrm{spiked}\;\mathrm{sample}}{\mathrm{Peak}\;\mathrm{area}\;\mathrm{in}\;\mathrm{post}\;\mathrm{spike}\;\mathrm{sample}}\times 100$$

(2) $$\mathrm{Matrix}\;\mathrm{factor}=\frac{\mathrm{Peak}\;\mathrm{area}\;\mathrm{in}\;\mathrm{post}\;\mathrm{spiked}\;\mathrm{sample}-\mathrm{Peak}\;\mathrm{area}\;\mathrm{in}\;\mathrm{unspiked}\;\mathrm{sample}}{\mathrm{Peak}\;\mathrm{area}\;\mathrm{in}\;\mathrm{neat}\;\mathrm{aqueous}\;\mathrm{sample}}$$

#### 3.5.5. Stability

The evaluation of stability was carried out to ensure that the steps taken during sample preparation and sample analysis, as well as the storage conditions, did not affect the concentration of the measured analyte. The stability of the stock and working solutions were tested with an appropriate dilution, while taking the linearity and measuring range of the detector into consideration. The stability was investigated in relation to storage conditions over time periods that were equal to those that were applied to the actual study samples. The stability of the stock solution and working solutions at −20 °C, and autosampler stability of the processed sample at injector or autosampler temperature (5 °C) for 48 h of the TCA cycle intermediates and internal standard were evaluated while using low (a maximum of three times the LLOQ) and high (close to the ULOQ) QC samples. They were immediately analyzed after preparation and after the applied storage conditions for 48 h. The QC samples were analyzed against a calibration curve, which was obtained from freshly spiked calibration standards, and the obtained concentrations as compared to the nominal concentrations. The mean concentration at each level should be within ±15% of the nominal concentration.

#### 3.5.6. Limit of Detection and Limit of Quantification

LOD was defined as the bottommost analyte concentrations giving a signal-to-noise (S/N) ratio of 3. The lowest standard concentration (Level 1) with acceptable accuracy and precision is considered to be LOQ. The test began with the serial dilution of the last extract of the samples.

#### 3.5.7. Comparison with NIST Reference Material

Commercially available standard-reference plasma (NIST SRM 1950) [22] was analyzed using the proposed validated method (*n* = 6 replicates) to evaluate the performance of the quantitative method. The concentration values for glycine, l-alanine, and serine were compared with the given standard reference values.

#### 3.5.8. Ethic Statement

Blood was procured from Finnish Red Cross Blood Service “punainen risti veripalvelu” (Helsinki, Finland) under license number 05/2019.

## 4. Conclusions

We present a comprehensively validated quantitative method for TCA cycle intermediates. The method was validated for human serum, plasma, kasumi-1 cell, and murine liver tissue, achieving high precision with the use of specific corresponding 13C isotope internal standards. The method can be used for high sample flow-through with minimized workload, due to the cost-efficiency, fast pretreatment, and short chromatography time. The method has the potential to be used in various research areas, such as nutrition, cancer, biomarker studies for different pathologies, and central carbon metabolism profiling as augmentation for metabolic flux analysis.

## Figures and Tables

**Table 1:** Accuracy, limit of detection (LOD) and limit of quantification (LOQ) results of TCA cycle intermediates.

Analyte	% Mean Accuracy ^a^ at Different Standard Concentration Level											LoQ (ng/mL)	LoD (ng/mL)
	L-1	L-2	L-3	L-4	L-5	L-6	L-7	L-8	L-9	L-10	L-11		
cis-Aconitic acid	85.12	80.24	86.54	81.91	94.99	85.73	99.17	104.58	101.65	99.12	100.07	11.72	8.79
Citric acid	103.38	86.48	90.49	93.10	95.52	104.64	101.99	103.79	103.69	101.25	98.51	122.07	12.21
Fumaric acid	96.42	115.90	97.13	109.16	97.60	101.16	99.09	95.49	95.91	98.73	102.65	23.44	17.58
Isocitric acid	102.28	106.47	135.45	138.34	116.83	103.05	91.41	83.08	86.35	95.54	114.94	29.30	14.65
Malic acid	101.33	86.40	82.40	83.07	101.73	104.68	103.53	105.99	104.21	102.07	97.29	6.25	3.13
Pyruvic acid	98.50	86.14	118.65	108.54	100.45	105.05	99.12	96.39	86.12	81.99	86.57	244.14	183.11
Oxoglutaric acid	99.61	101.24	125.54	108.43	99.51	101.72	99.24	98.11	98.44	98.38	98.45	122.07	61.04
Glycine	104.04	116.45	90.41	86.32	116.92	104.29	101.37	101.97	101.40	102.70	98.06	122.07	61.04
l-Alanine	94.24	109.23	101.87	101.99	105.04	103.66	102.49	99.33	97.90	92.59	91.66	122.07	12.21
l-Serine	97.38	100.70	117.07	107.11	100.20	101.90	99.76	100.26	99.10	91.96	96.08	29.30	2.93
l-Aspartic acid	97.84	95.56	121.37	102.72	101.81	101.74	102.04	102.50	99.70	89.49	92.91	11.72	5.86
l-Glutamine	98.28	99.04	104.86	104.54	104.57	105.26	101.34	99.12	89.24	75.12	63.02	122.07	6.10
l-Glutamic acid	97.16	88.41	108.66	102.11	102.84	96.74	97.30	99.39	100.14	100.13	98.48	29.30	2.93
l-Lactic acid	95.19	109.72	103.53	93.83	97.68	99.92	99.00	99.98	101.23	99.01	100.90	527.34	52.73
Succinic acid	98.98	101.25	104.02	95.32	98.26	101.43	100.23	103.12	102.37	98.57	96.42	23.44	11.72
d-Glucose	96.08	103.03	106.53	104.76	100.91	103.12	102.59	101.29	98.12	94.97	88.60	976.56	97.66

^a^: % mean accuracy is mean of accuracy of three calibration standard.

**Table 2:** Results of recovery, intra- and inter-day precision of TCA cycle intermediates in Serum and Tissue.

Analyte	Linearity Regression r^2^ Value ^a^	Intra-Day Precision of High, Medium and Low Concentrations, % RSD			Inter-Day Precision of High, Medium and Low Concentrations, % RSD			Mean Recovery Serum (%) ^b^	Mean Recovery Tissue (%) ^b^	Mean Recovery Cell (%) ^b^
		HQC	MQC	LQC	HQC	MQC	LQC			
cis-Aconitic acid	0.999	3.83	12.10	14.90	2.73	11.30	15.90	89.30	223.82	107.24
Citric acid	0.993	2.69	5.79	20.24	6.24	5.81	19.84	100.59	125.73	104.36
Fumaric acid	0.995	2.10	5.18	11.63	2.60	5.74	19.34	89.99	77.24	102.54
Isocitric acid	0.990	6.89	11.31	19.57	6.19	17.27	19.82	90.53	111.40	87.04
Malic acid	0.992	1.46	8.61	21.87	1.66	7.56	19.23	104.28	105.66	92.01
Pyruvic acid	0.990	3.36	4.06	14.51	10.86	8.91	12.06	85.00	132.64	91.48
Oxoglutaric acid	0.998	2.17	4.87	11.88	2.66	4.33	11.72	79.93	108.26	86.96
Glycine	0.996	3.20	4.84	17.26	3.95	6.59	19.06	105.34	111.00	108.73
l-Alanine	0.994	2.52	2.68	12.20	2.29	3.54	13.76	106.21	104.72	102.91
l-Serine	0.994	1.95	4.95	11.27	2.58	4.43	16.25	113.68	139.08	104.54
l-Aspartic acid	0.994	6.82	3.27	11.28	5.71	5.13	12.07	107.72	100.49	105.43
l-Glutamine	0.994	3.96	3.55	8.09	4.99	3.38	7.64	113.09	111.81	105.14
l-Glutamic acid	0.996	1.91	2.93	26.47	3.69	5.63	18.47	118.35	145.97	109.48
l-Lactic acid	0.992	1.34	4.93	18.97	1.43	4.30	17.93	103.81	79.26	107.18
Succinic acid	0.997	2.51	4.28	19.51	2.20	4.59	19.92	100.68	151.24	97.04
d-Glucose	0.996	1.92	2.72	3.86	2.25	2.69	3.74	96.36	101.98	100.45

^a^: Lowest r^2^ value across three calibration sets during validation; ^b^: Mean recovery of three QC levels.

**Table 3:** Results of stability for stock solution, working solution, and autosampler of TCA cycle intermediates.

Analyte	Stock Solution Stability, %		working Solution Stability, %		Autosampler Stability (%)	
	HQC	LQC	HQC	LQC	HQC	LQC
cis-Aconitic acid	89.13	90.01	87.52	91.02	88.28	89.23
Citric acid	90.12	95.01	91.05	96.12	91.29	94.35
Fumaric acid	100.20	94.25	101.39	95.01	99.80	93.91
Isocitric acid	109.62	111.47	108.52	114.23	112.59	114.80
Malic acid	97.34	86.01	98.10	86.50	98.64	85.56
Pyruvic acid	87.23	98.12	87.01	96.52	86.77	97.81
Oxoglutaric acid	98.99	91.23	96.52	90.34	97.90	89.36
Glycine	110.52	87.24	109.34	87.52	111.83	85.92
l-Alanine	89.25	113.24	89.34	112.24	88.04	115.25
l-Serine	90.52	102.34	91.04	101.11	91.85	101.58
l-Aspartic acid	92.00	107.32	93.34	108.00	92.73	108.68
l-Glutamine	86.23	100.12	87.54	99.01	85.45	101.81
l-Glutamic acid	95.12	109.12	93.12	108.92	94.87	107.59
l-Lactic acid	101.52	112.52	102.15	114.19	101.38	115.13
Succinic acid	97.85	112.52	98.14	113.00	98.07	114.61
d-Glucose	91.23	100.12	92.32	101.92	90.22	99.29

**Table 4:** Compound dependent parameters.

Analyte	Mass transition	Cone Voltage (eV)	Collision Voltage (V)	Ionization Mode
cis-Aconitic acid	172.97 > 128.9	22	8	Negative
Citric acid	191.1 > 110.94	16	10	Negative
Fumaric acid	115.1 > 41.00 & 70.94	32	6	Negative
Isocitric acid	191.1 > 155.00	20	12	Negative
Malic acid	133.1 > 114.91 & 71.00	34	10	Negative
Pyruvic acid	87.16 > 42.96	28	6	Negative
Oxoglutaric acid	145.1 > 100.91	22	6	Negative
Glycine	76.1 > 30.15	20	7	Positive
l-Alanine	90.1 > 44.15	20	10	Positive
l-Serine	106.1 > 60.5	23	7	Positive
l-Aspartic acid	134.15 > 74.05	29	13	Positive
l-Glutamine	147.1 > 130.1	26	8	Positive
l-Glutamic acid	148.1 > 130.1	22	9	Positive
l-Lactic acid	88.97 > 42.96	22	8	Negative
Succinic acid	117.05 > 73.00	22	12	Negative
d-Glucose	179.1 > 88.94	36	4	Negative

## Supplementary Materials

The following are available online at https://www.mdpi.com/2218-1989/10/3/103/s1, Figure S1: Structure of TCA cycle intermediates; Figure S2: Peak of TCA cycle intermediates; Table S1: List of Metabolites, Materials, Data of Recovery and Matrix effect.
