# Supplementary figures and images for: Simultaneous Measurement of Tricarboxylic Acid Cycle Intermediates in Different Biological Matrices Using Liquid Chromatography–Tandem Mass Spectrometry; Quantitation and Comparison of TCA Cycle Intermediates in Human Serum, Plasma, Kasumi-1 Cell and Murine Liver Tissue
Source: Metabolites. 2020 Mar 12;10(3):103. doi: 10.3390/metabo10030103 (PMC7143453; doi:10.3390/metabo10030103)

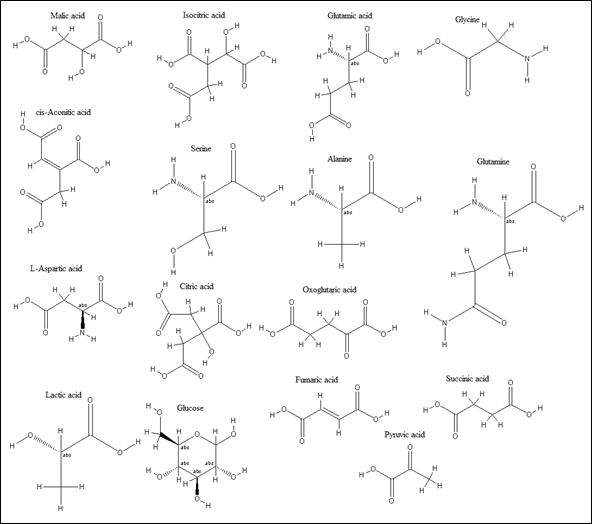

Supplement: Supplementary file 1 [file metabolites-10-00103-s001.zip › Figure S1 Structure of TCA cycle intermediates.png]

# TCA intermediate individual chromatogram

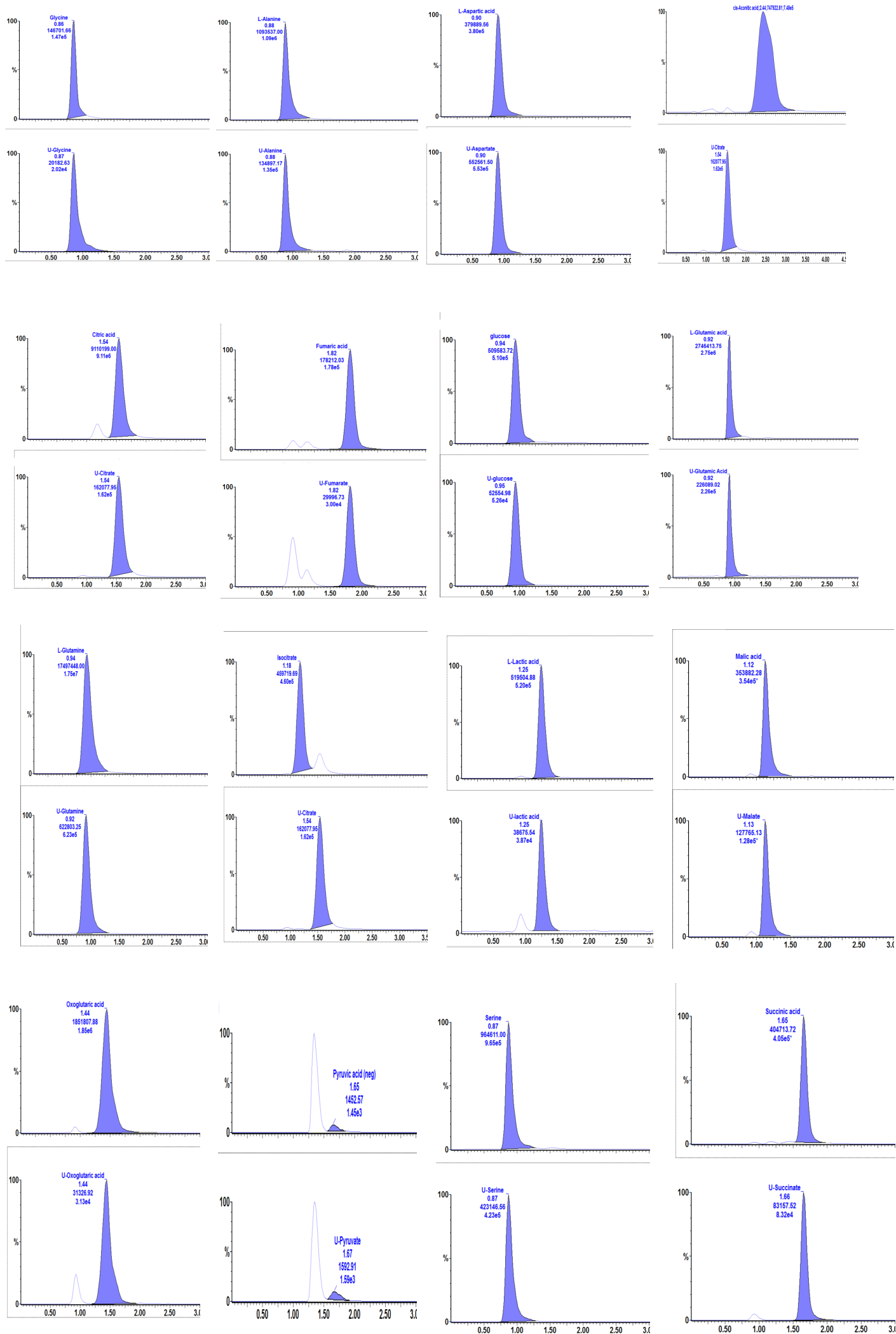

Supplement: Supplementary file 1 [file metabolites-10-00103-s001.zip › Figure S2 Peak of TCA cycle intermediates.pdf]
